# Touching the Lived Body in Patients with Medically Unexplained Symptoms. How an Integration of Hands-on Bodywork and Body Awareness in Psychotherapy may Help People with Alexithymia

**DOI:** 10.3389/fpsyg.2016.00253

**Published:** 2016-02-29

**Authors:** Joeri Calsius, Jozef De Bie, Raf Hertogen, Raf Meesen

**Affiliations:** ^1^Rehabilitation Research Center – Biomedical Research Center, Faculty of Medicine and Life Sciences, University of HasseltHasselt, Belgium; ^2^Department of Psychiatry, Ziekenhuis Oost-LimburgGenk, Belgium; ^3^Independent ResearcherHasselt, Belgium

**Keywords:** medically unexplained symptoms, alexithymia, touch, interoception, body awareness, psychotherapy, bodywork, phenomenology

## Abstract

Medically unexplained symptoms (MUS) are a considerable presenting problem in general practice. Alexithymia and difficulties with mental elaboration of bodily arousal are hypothesized as a key mechanism in MUS. In turn, this inability influences the embodied being and participating of these patients in the world, which is coined as ‘*the lived body*’ and underlies what is mostly referred to as body awareness (BA). The present article explores a more innovative hypothesis how hands-on bodywork can influence BA and serve as a rationale for a body integrated psychotherapeutic approach of MUS. Research not only shows that BA is a bottom-up ‘bodily’ affair but is anchored in a interoceptive-insular pathway (IIP) which in turn is deeply connected with autonomic and emotional brain areas as well as verbal and non-verbal memory. Moreover, it is emphasized how skin and myofascial tissues should be seen as an interoceptive generator, if approached in the proper manual way. This article offers supportive evidence explaining why a ‘haptic’ touch activates this IIP, restores the myofascial armored body, helps patients rebalancing their window of tolerance and facilitates BA by contacting their bodily inner-world. From a *trans*-disciplinary angle this article reflects on how the integration of bodywork with non-directive verbal guidance can be deeply healing and resourcing for the lived body experience in MUS. In particular for alexithymic patients this approach can be of significance regarding their representational failure of bodily arousal.

## Introduction

Medically unexplained symptoms (MUS) are estimated to be the presenting problem for 35% up to 64% of new patients in general practice (Jackson and Passamonti, 2005; Steinbrecher et al., 2011) and for 37–66% of new outpatients at specialist medical clinics (Nimnuan et al., 2001). The term MUS has received a lot of criticism, yet, recent diagnostic ‘improvements’ such as ‘functional somatic syndromes’ or ‘somatic symptom disorder’ in DSM-V still fail to offer a better etiological understanding and framework for therapy. This is especially the case for DSM-V (Frances, 2014; Vanheule, 2014; Vanheule et al., 2014). Therapeutic tools are not really based on a sound pathophysiological understanding and both pharmacological and non-pharmacological treatment outcomes are often disappointing (Kleinstäuber et al., 2014; Van Dessel et al., 2014).

On the other hand there is an increasing amount of research that shows the importance of personal history, attachment, and trauma (Van der Kolk, 1994, 2014; Taylor et al., 2000; Wearden et al., 2005; Buffington, 2009) where affect regulation seems to be a key element in understanding MUS (Waller and Scheidt, 2006). It even offers concrete implications for therapy since the ability to regulate negative or positive inner arousal seems to be difficult or even absent in patients with MUS, whether or not related to trauma (Kooiman et al., 2000, 2004; Van der Kolk, 2005, 2009; Grabe et al., 2008; Huber et al., 2009; Bogaerts et al., 2010). From a psychiatric and psychological point of view this inability is conceptualized as a personality trait called alexithymia (Sifneos, 1973; Bermond et al., 2006). Numerous studies have identified alexithymia and the absence of mental elaboration of bodily arousal as a key mechanism in the etiology or maintenance of MUS such as chronic fatigue syndrome, fibromyalgia, non-cardiac chest pain, irritable bowel syndrome, and to some extent panic attacks as well as medical and psychiatric disorders (De Gucht and Heiser, 2003; De Gucht et al., 2004; Herbert et al., 2011; Vanheule et al., 2011a,b; Meganck et al., 2013). To some extent alexithymia can be seen as the opposite of mentalization which refers to the ability to explore and express one’s own bodily sensations and feelings, as well as the emotional presence and intentions of others. Mentalization-based treatments have proven to be efficient for different populations of patients with psychosomatic or psychiatric problems. However, a large group of patients with MUS keeps presenting in therapy, making no progress and leaving the therapist frustrated looking for novel or alternative tools.

This present article will try to clarify in a trans-disciplinary way how an integrated psychotherapeutic approach of hands-on bodywork and body awareness (BA) may be an interesting and refreshing approach for these hard-to-treat patients. Although this kind of integration is distinctive for what is mostly referred to as body (oriented) psychotherapy, body mind therapy or somatic psychotherapy, it is often overlooked or neglected in mainstream psychotherapy (Totton, 2005; Heller, 2012; Cipolletta, 2013; Cornell, 2015; Geuter, 2015). To avoid conceptual misunderstanding or school-dependent vocabulary, this article will use ‘experiential bodywork’ (EBW) as an umbrella term for those body mind therapies using an explicit hands-on approach, often in combination with movement-based strategies for emotional awareness and expression. So in EBW the therapist works with BA and touch to bring patients in contact with their inner world and to facilitate interpersonal dynamics. Being a touch-based experiential approach, EBW incorporates hands-on techniques to work specifically on the patients’ body, which will be detailed in this article as the myofascial system. Basically EBW is a phenomenological approach, focussing on the lived body experience of the patient. Although BA therapies in general are often lacking solid scientific evidence (Courtois et al., 2015), a compelling body of research shows how being aware of your body is essential in normal development and health outcomes (Fuchs, 2001; Gard, 2005; Osborn and Smith, 2006; Bullington, 2009; Fuchs and Schlimme, 2009; Payne, 2009; Zeiler, 2010; Fogel, 2011; Cipolletta et al., 2014). Especially the hands-on feature of EBW is hypothesized to be a powerful aspect of the therapeutic process but is –however– not researched well enough in relation to MUS.

The hypothesis explored in this article is that integrating bodywork in psychotherapy is a useful strategy for MUS, specifically in relation to alexithymia. Moreover, a touch-based ‘*myofascial*’ entry point can have an added value on BA as a therapeutic outcome and on the lived body experience in general. To support this hypothesis, the role of interoception in relation to BA, affect regulation and psychosomatics will need to be discussed. So after firstly situating how MUS and alexithymia are related to BA processing, this article will secondly elaborate in detail on the significance and specificity of interoceptive brain-pathways to BA. Finally this will lead to possible underpinnings for a more innovative hypothesis in which we explore how myofascial tissue dynamics, as related to hands-on bodywork, influence BA-processing and how this can serve as a plausible rationale for a body integrated therapeutic approach of MUS.

## Part I: Alexithymia, MUS and BA Processing

In their review, Rief and Broadbent (2007) describe how different etiological models for MUS have developed over the last two decades. Most of these models point at the maintaining or causal influence of cognitive factors such as symptom attribution, illness belief, attention, perception, and memory bias. Besides these elements, the importance of attachment style and personality factors, especially those traits that are involved in emotional regulation, are stressed. The concept of alexithymia as the inability to explore one’s inner world is often used to understand how MUS can develop. Since Sifneos (1973) introduced the concept of alexithymia there has been an exponential interest in its relationship with psychopathology although a unanimous explanation is lacking. Nevertheless alexithymia seems to be one of the important underpinnings in understanding how stress-inducing factors in one’s personal life can lead to MUS or somatization in general (De Gucht and Heiser, 2003).

Leaving the mere symptomatic level and trying to grasp the more etiological dynamics of MUS empirical research has shown that what is called somatization should be divided in two categories, i.e., presenting and functional somatization (De Gucht and Heiser, 2003; De Gucht et al., 2004). ‘Presenting’ somatization is considered as secondary to psycho-emotional distress where in functional somatization, the somatization itself is seen as a primary phenomenon, typically characterized by unexplainable symptoms. In the latter somatization can be understood as a somatic equivalent of an anxiety disorder or depression whereby the personality trait alexithymia could be a significant factor in the appearance of the unexplainable symptoms (Verhaeghe and Vanheule, 2005). So in functional somatization, the bodily distress comes *instead* of the mental phenomenon of anxiety because the patient is unable to explore his arousal and to elaborate on it mentally using language, metaphors, or phantasies.

This explanation is in line with numerous observations in trauma patients who are unable to connect the traumatic event with a suitable narrative to explain what they went through, which in fact seems crucial for recovery: “*Only when they (traumatic memories) have been fully assimilated and assigned to the hippocampal timeline can they become integrated and experienced as* ‘*just a memory*’ *in the past”* (Payne et al., 2015). Research also strongly stresses the bottom-up mortgage that trauma poses on normal processing of intense emotions and shows how dysfunctional bodily processes involving breathing, dystonia, immunology, and digestion are involved (Guilbaud et al., 2003; Levine, 2005; Ogden et al., 2006; Siegel, 2009; Van der Kolk, 2014). In particular the role of the autonomic nervous system has led to several models of psychosomatic dysfunction or trauma related disorder such as the visceral brain-body transfer (Cameron, 2009), the polyvagal theory (Porges, 2009), the neurovisceral integration (Thayer and Brosschot, 2005; Cameron, 2009), the window of tolerance (Ogden et al., 2006) or more recently the preparatory set (Payne and Crane-Godreau, 2015). In essence all these models point at the significant influence of a deeply disturbed autonomic nervous system with severe consequences for all afferent and efferent processing. In this regard, especially interoception is essential and seems to be a key element in what leads to body- and self-awareness (Cameron, 2001, 2009; Craig, 2003, 2011), which in turn is the quintessence of EBW.

## Part II: Brain Pathways of Body Awareness and Interoception

In phenomenology an individual is conceived as the perspectival origin of his experiences, behavior, and thoughts and seen as the center of self-awareness, object-experience and meaning bestowing (Stanghellini, 2009). This embodied presence in the world is coined as ‘*the lived body*’ and underlies what is mostly referred to as BA. The lived body is seen as the phenomenological fundament of being-in-the-world and serves at the same time as the key entry point to the self and the world: we literally stand out [latin: existere] in our world and so we exist. Gyllensten et al. (2010) describe the concept of the embodied identity as related to living in the body and living in relation to others and society. Here, BA fits in embodied identity: being aware of my bodily being in the world grounds my experience of who I am as an embodied identity. So my identity is not just a conceptual understanding of my self, it is always and foremost a bodily being-related-to and a being-embedded-in the world, as Merleau–Ponty has grounded the concept of embodiment (Carman, 2008). In relation to embodiment, BA is the awareness of embodiment as an innate tendency of our organism for emergent self-organization and wholeness (Mehling et al., 2011). Fogel (2009) describes BA as the ability to pay attention to ourselves, to feel our sensations, and movements online, along with the motivational and emotional feelings that accompany them, in the present moment, without the mediating influence of judgmental thoughts. Mehling et al. (2011) sees BA as the subjective, phenomenological aspect of proprioception and interoception that enters conscious awareness and is modifiable by mental processes including attention, interpretation, appraisal, beliefs, memories, conditioning attitude, and affect. In that way interoception is central to BA, next to body scheme, existing of exteroception and proprioception. Besides typical examples of interoceptive sensations like cold, itch, tickle or visceral urgency, also tensed muscles or muscle tone are processed interoceptively (Fogel, 2009).

Since Craig (2009) coined interoception as information coming from all over the body different authors propose interoception as the neuroanatomical pathway leading to the emergence of BA (Vaitl, 1996; Critchley et al., 2004; Walusinski, 2006). Interoception is triggered when changes occur in homeostasis, registered by different kinds of receptors in, e.g., skin, joints, muscles, internal organs, and is supported by two main tracts, the parasympathetic and the sympathetic afferents (Vaitl, 1996). An important characteristic of the ascending afferent sympathetic route is its connection with the parasympathetic route (Walusinski, 2006). This link allows integrated maps of our bodily conditions being generated (Damasio and Carvalho, 2013). The sympathetic afferents, on the one hand, make a monosynaptic connection onto lamina I, whereas the fibers from the parasympathetic pathway project to the nucleus of the solitary tract (Craig, 2003), which integrates general information on arousal, sexuality, and nourishment (Walusinski, 2006).

In primates, lamina I output neurons have three different projections sites: the spinal autonomic nuclei, the parabrachial nuclei together with the periaqueductal gray and hypothalamic portion and finally there is the cortical higher-order posterior part of the ventromedial nucleus of the thalamus, situated in the forebrain (Craig, 2002, 2009). The complementary parabrachial nuclei and the periaqueductal gray are the most important integrators on brain stem level for all the homeostatic afferent information necessary for survival (Craig, 2003). The output neurons of the nucleus of the solitary tract solely have two different projection sites, located in the same regions as the sympathetic afferent route: the parabrachial nuclei and the ventromedial nucleus of the thalamus. Only in primates this afferent information can bypass the brainstem directly via a pathway directly going from lamina I and the nucleus of the solitary tract to the thalamus. The other route, where afferent homeostatic information is first conducted to the parabrachial nuclei in the brainstem before arriving at the thalamus, is found in sub-primates. In turn, the thalamic nucleus projects all interoceptive afferent information, coming from the body, on the posterior insula. This brain-pathway differs significantly from the somatosensory cortices that receive exteroceptive input, explaining the distinct neuroanatomical basis of interoception and exteroception (Craig, 2002).

### The Role of the Insula in Body Awareness

**FIGURE 1:**
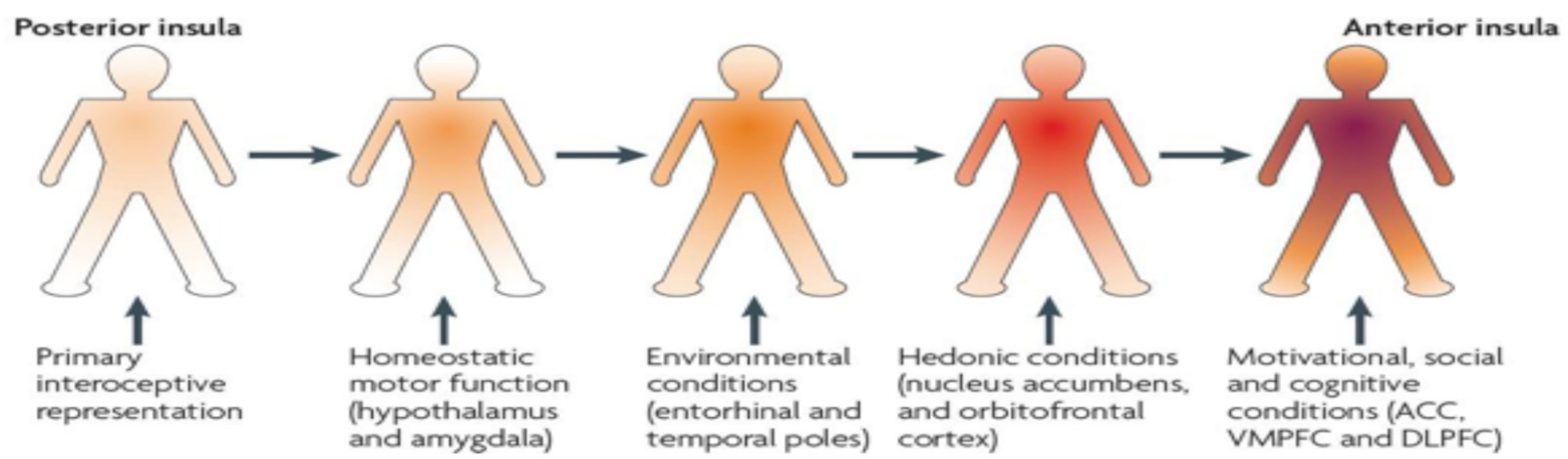
**Posterior-to-anterior gradient of awareness proposed by Craig (2009)**.

As shown in **Figure 1**, a progressive integration of the afferent information via a posterior-to-anterior sequence takes place in the insula (Craig, 2009; Simmons et al., 2013). So the afferent information emerging from the body finds its way from the posterior, over the mid, to the anterior insula. This running from the posterior insula, where a first-order representation is mapping the physiological condition of the body as an ‘*interoceptive image*,’ leaves the mid insula as an important integrative zone on the cortical level (Craig, 2010, 2011). From the mid-insula, all information goes to the left or right anterior insula, where a motivational, social, cognitive, and hedonic dimension is added and an ultimate re-representation or meta-image is made (Craig, 2009; Berlucchi and Aglioti, 2010; Herbert and Pollatos, 2012). Craig (2004, 2011) states that the right non-dominant anterior insular cortex is the most important structure in detecting our inner feelings.

Different authors also suggest a lateralization in the hemispheres, based on the type of stimulus that is presented (Cameron and Minoshima, 2002; Pollatos et al., 2007; Craig, 2009). This determines whether the re-representation will occur in the right insula (when a sympathetic, negative, or energy expenditure stimulus is presented) or left anterior insula (when a parasympathetic, positive, or energy-nourishing stimulus is presented (Craig, 2009, 2011). In relation to MUS, a disharmonious and prolonged activation of the sympathetic over the parasympathetic seems to be associated with disinhibited defensive circuits that in turn can be pathogenic for psychosomatic dysfunctioning (Thayer and Brosschot, 2005).

When afferent information becomes more complex during the posterior-to-anterior gradient in the insula, the posterior insula –suggested to have an important role in triggering the pain matrix network (Porges, 2009; Isnard et al., 2011; Lutz et al., 2013)– produces a first-order, objective, non-contextual representation, while the anterior insula is related to more subjective feelings. Craig (2009) notes that this process only takes place when attention is focused on the presented stimulus as explicated in the definition of BA. In this way, interoception can be modulated by changing attention, emotions, or mood (Bushnell et al., 2013; Häfner, 2013). More specific, the right anterior insula is seen by Craig (2009) as the sole structure that creates subjective feelings, resulting from the body, into awareness through a second order re-representation and which makes it the neural substrate for body and self-awareness (Tsakiris et al., 2007; Khalsa et al., 2008, 2009; Berlucchi and Aglioti, 2010; Tsakiris, 2010). This finding of the right anterior insula as dominant for interoception is supported by different studies (Critchley et al., 2004; Pollatos et al., 2005, 2007; Walusinski, 2006; Critchley and Seth, 2012; Herbert and Pollatos, 2012; Seth, 2013). Further, the anterior insula is influenced by top-down predictions from high level cortical areas such as the anterior cingulate cortex or the prefrontal cortex and in turn, provides descending interoceptive predictions to efferent systems that guide information for autonomic reflexes (Gu et al., 2013; Seth, 2013). Up to this point of the anterior insula, it can be concluded that this interoceptive-insular pathway provides the neural correlate for BA.

### The Interoceptive Pathway and the Limbic System

Subsequently, the interoceptive-insular pathway proceeds from the anterior insula to the closely linked emotional activating brain sites: the anterior cingulate cortex, hypothalamus, amygdala, and the orbitofrontal cortex that adds a hedonic-affective loading to the felt sensation of the body (Craig, 2003; Bechara and Naqvi, 2004). The anterior cingulate cortex and the anterior insula serve together as two complementary limbic regions in virtually all emotions (the latter can be seen as the limbic sensory cortex and the first as the limbic motor counterpart). This anterior cingulate cortex provides feelings arising from the body with a motivational element, while the insula adds an emotional element (Craig, 2002). The latter moderates feelings and awareness and the first does the same for motivations and behaviors (Craig, 2011). The orbitofrontal cortex and the right anterior insula are the only brain regions where the size of brain area has a direct association with interoceptive awareness which might suggest strong evidence for these structures to play a role in interoception (Craig, 2004).

So in origin also emotions are formed via this interoceptive-insular pathway, based on arousal emerging from the body that supports homeostasis. This is adherent to the James–Lange theory of emotions that states that a frightening stimulus leads to physical reactions (e.g., sweating, heart pounding), leading eventually to an emotion (fear) which is the result of the interpretation of these bodily states and not of a cognitive top-down analysis of the frightening stimulus. The interoceptive-insular pathway also supports the ‘somatic marker’ hypothesis of Damasio (2003) suggesting that higher-order decision-making relies strongly on bodily interoceptive input. The description of the interoceptive BA-pathway by Craig is similar to Cameron (2001) and Walusinski (2006), but Cameron states that a third major pathway –i.e., the enteric nervous system– has to be distinguished and adds that the sympathetic afferent fibers also project on lamina’s V, VII, and VIII. Also Damasio’s (2003) vision is highly congruent with Craig’s, but there are significant differences. Although Damasio (2003) describes the sympathetic and parasympathetic route in a similar way, he puts more emphasis on the hypothalamus, which is regarded as the homeostatic motor center of the diencephalon, and the periaqueductal gray, viewed as the homeostatic motor center of the mesencephalon (Craig, 2003). Following Damasio (2003), the ability to experience feelings is also derived from the brainstem and probably from the somatosensory cortices so he concludes that a more modular role –instead of a generative one– should be attributed to the insula.

## Part III: Integrating Body Awareness from a Myofascial Perspective in Experiential Bodywork

As one could wonder why a detailed analysis of the different views on BA-pathways is necessary for the aim of this article, some reflections have to be taken into account. First of all, a profound insight in the interoceptive underpinnings of BA is a requisite for understanding which structures –from peripheral receptors to central brain areas– are involved in the gradual process of becoming aware of ones bodily self. Although there seems to be some dissonance on the degree of involvement –especially the insula as the sole integrator and generator of BA– there is fair agreement on the common ground in BA-pathways. Literally all bodily tissues and regions are to some extent tuning into the interoceptive-insular pathway via a parasympathetic, sympathetic, or enteric route. In this hierarchical bottom-up processing there seems to be a significant role of the insular level regarding the emergence of BA and its embedding in the limbic emotional network. Secondly, it is important to know that besides this interoceptive-insular pathway, other functional systems like the immunological and visceral, are contributing to BA together with extero- and proprioceptive processing. Altogether, this strongly emphasizes how BA is above all a bottom-up ‘bodily’ affair, in line with emotional theories of James-Lange or Damasio. Although this could sound intuitively right, it is only due to rigorous research on processes such as BA that the cognitive hegemony of the previous decade has been corrected in favor of ‘sub-cortical processing’ (Payne et al., 2015) and this has substantial consequences for therapy as will be explained further on. Finally, a detailed analysis of BA-pathways gives a preliminary explanation for clinical observations or psychological and phenomenological concepts –such as *the body as source of tacit knowledge*– that are common among bodyworkers and psychotherapists, as will be elucidated.

### Myofascial Tissue and BA

Based on this detailed description of BA-pathways and aiming at a therapeutic transfer in the end, we are urged to look again at the definition of BA where Fogel (2009) highlighted the *online* registration of what is experienced without mediation by cognitive processes, while Mehling et al. (2011, 2009) stresses that BA is in particular about a *conscious* processing of the re-representation of all bodily arousal arriving at the insula. To this extent BA can be understood as the phenomenon emerging at the level of the anterior insula referring at the different authors who theorize the posterior to anterior insular gradient as a shift in becoming self-aware. However, as this article intended to focus on possible clinical implications it should be reminded that the difficulty or even inability of alexithymic patients suffering from MUS lies especially in registering, exploring and finally putting into words what they experience on a bodily emotional level. It is in particular at this point that the added ‘hands-on’ value of EBW can come into play as its primary entry-point –the myofascial tissue– offers a unique gateway to the interoceptive brain-body pathways.

However, this uniqueness is twofold in that sense that it is situated on a more etiological as well as a therapeutic level. As extensively described, the interoceptive-insular pathway consists of ‘whole body’ sensory input, which includes *every* myofascial tissue structure. Indeed, several studies (Stecco et al., 2007; Benjamin, 2009) not only show how myofascial tissue is richly innervated by unmyelinated poly-modal C-fibers –by Schleip (2003a,b) labeled as interstitial myofascial tissue receptors (IMTR)– but they also indicate how these IMTR react to a specific manual approach, described as tangential or slow melting pressure. Moreover, the myofibroblasts, which are abundantly present in myofascial tissue, are driven by IMTR and are found capable to generate a so called fascial pretension (Schleip, 2003a,b; Langevin and Huijing, 2009; Abu-Hijleh et al., 2012; Oschman, 2012; Levin and Martin, 2012; Schleip and Jäger, 2012). Although this tensional force of fascia can operate autonomously from muscle tone (Schleip et al., 2005, 2012; Masi and Hannon, 2008; Schleip, 2011), it is thought that in endured conditions it can lead up to a fascial stiffness due to its sensitivity for over-activation of the autonomic nervous system or stress (Liptan, 2010; Donadio et al., 2012). Key link in the transmission between sympathetic over-tuning and myofibroblast proliferation resulting in myofascial tissue stiffness seems to be TGF-béta 1 (transforming growth factor). In addition to this more fascia-related influence of stress, it is also known that muscle load *per se* is perceived as interoceptive input based on contractile as well as metabolic tissue sensitivity. This is why Craig (2002) speaks of a continuous drive of a variety of regional and whole-body adjustments to muscular work being processed as interoception. In addition specific receptors –called human tactile C-fibers– are identified as tuning the interoceptive-insular pathway when responding to ‘light sensual touch’ (Olausson et al., 2002, 2008, 2010; Björnsdotter et al., 2010; Ackerley et al., 2014; McGlone et al., 2014). Furthermore, this synopsis is consistent with research showing how insular activity is linked with human touch and attachment (Taylor et al., 2000; Bartels and Zeki, 2004; Field et al., 2009; Lovero et al., 2009; Field, 2010; Herbert and Pollatos, 2012).

On a more clinical level a lot of this research echoes what Reich (1972, 1973) saw from a empirical-intuitive basis in his patients as an armoring of the body which he defined as the experience-dependent development of a protective shell of muscle tension grown over time in response to a history of threat, anxiety and trauma. To this *muscular armor* he (Reich, 1973) attributed characteristics such as it stiffens the coordination of the segments of the body, reduces the postural repertoire, inhibits respiration, and diminishes the perception of what goes on in the body, which are very recognizable in MUS. This idea of the body as a blueprint or expression of personal experiencing and psychodynamic coping is also more and more supported from neuro-developmental as well as psychological perspectives. In that sense, neuromuscular and autonomic nervous systems are thought to encode patterns of early object relations, so that there may be a long term autobiographical memory of a pathological internal object relation, that becomes the unconscious working model (Schore, 2001). In addition it is stressed that attachment which is basically described as mental pattern, is also held in place by chronic physical tendencies reflective of early attachment pattern (Ogden et al., 2006). Taken together, it could be summarized that the whole myofascial substrate including muscle, fascia and skin –as detailed in this section– is referring at the concept of our patients armored body and should be seen as a interoceptive generator, if approached in the proper manual way.

### Why is Interoception so Paramount?

By now we know that (a) the whole skin and myofascial tissue functions as an interoceptive sensitive substrate if approached properly, (b) the interoceptive-insular pathway is deeply connected with our autonomic and emotional brain areas together with the neuro-anatomical correlates of verbal and non-verbal memory storage and (c) this interoceptive-insular pathway not only serves as the substrate for the emergence of BA but is also credited as crucial in normal or psychosomatic functioning. Herewith a theoretical basis is offered for what in EBW is often referred to as a ‘*listening touch*’ (Fogel, 2009), a ‘*limbic touch*’ (Olausson et al., 2002; Van der Kolk, 2014) or a ‘*medium for haptic perception*’ (Turvey and Fonseca, 2014). If earlier BA was described as the quintessence of EBW, a threefold logic constitutes its keystone and can be synthesized as follows: (1) interoception is crucial in psychosomatic disorder and somatization, (2) the interoceptive-insular pathway is the central correlate of what phenomenologically is called BA and (3) myofascial tissue is not only our largest interoceptive organ but can react in a dysfunctional or even pathological way to endured stress as in a loaded emotional context or trauma.

So leaving the mere neuroanatomical analysis and resuming a clinical and therapy oriented stance, BA can now be seen as a privileged entry-point to psychosomatics in general and MUS specifically. Reminding now how alexithymia links with MUS brings up the question if BA as a therapeutic goal in EBW can offer the hypothesized added value. Here a further differentiation of BA, conceptualized by Fogel (2009, 2011), offers a possible step stone in fine-tuning how a specific manual approach helps alexithymic people (re-)contacting and exploring their inner-world. Fogel (2009, 2011) uses the term ‘*embodied self-awareness*’ to describe the ability to be in the subjective emotional present and to explore the intricacies of senses, movements, and emotions in relation to others and the world. Hereby, embodied self-awareness is labeled as spontaneous, creative, and open to change, concrete and lived in the present moment and most of all characterized by its non-verbal, *trans*-lingual nature.

All of these features are congruent with the pre-reflective and non-verbal nature of BA as a tacit but experiential primordial contact with the world. Besides the embodied outward relation to the world, there is also an inward relation that has been extensively analyzed and paraphrased as the body being a source of knowledge or wisdom (Wilde, 2003; Fuchs and Schlimme, 2009; Stanghellini, 2009; Gyllensten et al., 2010). Here the individual who listens and tunes in to his body can learn and experience an inner approval or meaning bestowing which is sometimes labeled as a felt sense (Gendlin, 1969; Gendlin and Olsen, 1970). From a phenomenological perspective, it has been studied why in particular this ability of BA as tuning-in and feeling the self as an embodied intelligence, is often not available in people with chronic illness or pain (Price, 1993; Bullington, 2009; Calsius et al., 2015). When Fogel (2009) states that embodied self-awareness does not require language for its expression and exists prior to language, he is referring at the developmental processing of its key neurobiological correlate, namely the amygdala. In line with Craig, Fogel sees the interoceptive-insular pathway as the backbone of BA but although equally stressing the exclusive interest of the insular capacity to re-representation, he links the lower and limbic brain structures to embodied self-awareness. A profound therapeutic consequence of this is the use of *evocative language* (Fogel, 2009) or *imagery playing-out* (Payne and Crane-Godreau, 2015; Payne et al., 2015) to enter, explore and express embodied self-awareness in patients. In contrast to regular language that is more under cognitive control and censorship, evocative language and imagery are resonating the felt experience ‘as it appears in the present moment’ (Gendlin, 1969; Gendlin and Olsen, 1970; Bloom, 2006; Fogel, 2009). Besides spontaneous words that are capturing this resonance or felt sense also metaphors, images, dreams, poetry, music, drama, or meditation are examples and from a more psychoanalytical angle, free association is evocative language alike.

As described earlier, it is precisely this ability to embodied self-awareness that is lacking in alexithymic people and makes them vulnerable to psychosomatic dysfunction as a wordless gap lies between the level of bodily arousal and the transfer into language or imagination. So working with embodied self-awareness in therapy could be meaningful as EBW is specifically aiming at closing this gap. Herewith we are arriving at the central question of this article on how a manual, touch-based approach can be a unique entry-point.

### Touching the Body, Moving the Words

When Vanheule et al. (2011b) approach alexithymia from a psychoanalytical perspective they describe it as a representational failure of bodily arousal and tension when people do not understand what is going on in their body while experiencing for example stress or trivial somatic signals. Subsequently they point at the importance of the progressive and patient-fit process of finding words to describe the sensations and feelings, i.e., symbolisation. At the same time they stress that “*treatment should not focus on the mental representational deficit as such, but on the underlying experiences, with which the patient fails to deal mentally*” (Vanheule et al., 2011b, p. 91). As psychotherapists they propose a three-step logic wherein the un-mentalized experiences are facilitated into an autobiographical narrative. Although a psychoanalytic approach would intuitively not seem to aim at the same therapeutic goals, there appears to be quite some resemblance with therapies in EBW.

Given the use of a ‘listening,’ ‘haptic,’ or ‘limbic’ touch EBW not only helps the patient tuning into the interoceptive-insular pathway and rebalancing the window of tolerance to an optimal arousal zone –mostly by silencing the sympathetic overdrive– but specifically facilitates the ability to become body aware and to contact one’s own bodily inner world. In short, EBW creates an optimal momentum for embodied self-awareness and invites or even provokes evocative language. In particular, the limbic load of the interoceptive-insular pathway is hypothesized to affect the experiential BA-process during the hands-on approach. The combination of light and tactile manual bodywork with non-directive verbal guidance is triggering the dyadic bodily resonance described by several authors as being deeply healing, restoring and resourcing (Totton, 1998, 2005; Levine, 2005; Bloom, 2006; Ogden et al., 2006; White, 2006; Heller, 2012; Cipolletta, 2013; Van der Kolk, 2014; Cornell, 2015; Payne et al., 2015). Trying to grasp the underlying mechanism of this beneficial process of EBW, research mostly points at optimizing and rebalancing of a secure attachment (Ogden et al., 2006), at opening up the social engagement system (Porges, 2009), at engaging and integrating preverbal memories (Bloom, 2006), at befriending the body, which is a metaphor for allowing and naming the physical sensations beneath the emotions (Van der Kolk, 2014) or at biological completion (Payne and Crane-Godreau, 2015; Payne et al., 2015). The latter authors are putting forward a hypothesis for understanding body mind therapies as normalizing what they call a preparatory set, which is basically a unitary trauma response based on affect, posture and muscle tone, autonomic state, attention, and expectation and which they call the core response network. Although all of these authors are to some extent drawing on neurophysiological frameworks, they ultimately point at the quintessence of becoming self-aware from within the body, which is convergent with the realm of phenomenological BA-psychotherapies and EBW. Wilde (2003) rephrases this more explicitly focussing on the experiential level and using BA to enter the lived body as contacting ‘*a silent partner of informant of embodied knowledge.*’ Interestingly, Payne et al. (2015) point at the same two principal ways how what they name as body-mind therapeutic or educational systems can work, by becoming aware and by biological completion such as trembling, crying or flushing in which the originally obstructed load is enabled to complete itself.

As proposed in this present article, in EBW these two principals are intertwined firstly because the listening touch in EBW –whether hands on body work is combined with verbal exploration at the same time or more in a phased protocol– is offering a concrete way-in to the patients inner world of sensations and emotions while serving as a tangible support for BA. In particular for alexithymic patients, this guidance can be of significance regarding their representational failure of bodily arousal and tension. Secondly, given the limbic load of touch in EBW, the therapeutic frame is now expanded with an inside-out bodily experience of a safe, attentive and authentic presence, crucial for holding and containment. Although not required, this can facilitate a cathartic process –comparable with Payne’s idea of biological completion and Reichs earlier thoughts on a break-through– where the patient is guided in expressing or acting-out in a more evocative or bodily language what we earlier called un-mentalized material. This seeking for words of symbolization puts the patient (back) at the perspectival origin of his experiences, behavior, and thoughts so he can learn once again to trust his body as a source of information and knowledge and create the necessary counter-weight to correct his often hyper-cognitive ways of coping with stressful situations.

## Conclusion

Body awareness as the ability to contact and feel one’s body from within is considered a key feature in normal functioning and general health. On the other hand alexithymia weighs on the etiology of psychosomatic processing and MUS in particular. In that way, working with the inability to mentalize bodily arousal, tension, and inner feelings together with its representational failure has a pivotal role in therapy with MUS-patients. Given the often disappointing outcomes of verbal psychotherapies as well as somatic therapies, this article reflected on how hands-on bodywork influences BA, helps patients to mentalize and express what they experience and legitimizes a more body integrated psychotherapeutic approach of MUS.

First of all interoception was detailed as paramount for understanding the phenomenology of BA and grounding its possible therapeutic implementations. Intertwined with the neurophysiological pathways of interoception, the interstitial myofascial tissue receptors and human tactile C-fibers are thought to explain –at least partially– how touch can play a unique role in therapy other than its most known extero- and proprioceptive functioning. Key elements here are that (a) the whole skin and myofascial tissue serves as an interoceptive sensitive substrate if approached properly, (b) the interoceptive-insular pathway is deeply connected with our autonomic and emotional brain areas together with the neuro-anatomical correlates of verbal and non-verbal memory, and (c) this interoceptive-insular pathway not only constitutes the substrate for the emergence of BA but is also credited as crucial in normal or psychosomatic functioning. Hereby the body moves from its previous peripheral and secondary position in psychotherapy to a more central spot whereby research has shown how a continuous drive of regional and whole-body adjustments to muscular ‘myofascial’ bodywork is being processed as interoception. In short, the (myofascial) body cannot be seen anymore as solely a passive ‘thing’ that only reacts on psycho-emotional load but is an interactive and instructive ‘agens’ that tunes feelings, thoughts, and the lived body in general.

Subsequently not only verbal BA interventions can be seen as a privileged entry-point to this interoceptive realm but especially the use of ‘haptic’ or ‘listening’ touch can offer patients with MUS a concrete way-in to their inner world of experiencing while serving as a tangible support. It puts these patients once again at the perspectival origin of their experiences and helps to trust their body as a source of tacit knowledge and counter-weight on the often hyper-cognitive ways of coping with stress. Co-using evocative language and movement to explore and express their inner realms, helps bypassing the alexithymic gap of these patients and makes the embodied self-awareness resonate ‘as it appears in the present moment.’

From a *trans*-disciplinary angle this article contributed to a more sound understanding of experiential bodywork as an added value in psychotherapy in MUS. Although further research and clinical outcome studies are essential to legitimize this intriguing relationship between bodywork and psychotherapy, a compelling amount of state of the art research seems to confirm what intuitively and empirically is known since the hay-days of psychotherapy: the body cannot be left out when entering and treating the psychosomatic circle.

## Author Contributions

All authors listed, have made substantial, direct and intellectual contribution to the work, and approved it for publication.

## Conflict of Interest Statement

The authors declare that the research was conducted in the absence of any commercial or financial relationships that could be construed as a potential conflict of interest.
